# Physicians’ experiences and perceived challenges working in an emergency setting in Bharatpur, Nepal: a qualitative study

**DOI:** 10.1186/s12245-022-00466-w

**Published:** 2022-11-08

**Authors:** Kristoffer Lund Hansen, Åsmund Bratholm, Manohar Pradhan, Søren Mikkelsen, Louise Milling

**Affiliations:** 1 The Prehospital Research Unit, Region of Southern Denmark, Institute of Regional Health Research, University of Southern Denmark & Odense University Hospital, Odense, Denmark; 2 Department of General Practice & Emergency Medicine, College of Medical Sciences Teaching Hospital, Bharatpur, Nepal; 3 Mobile Emergency Care Unit, Department of Anaesthesiology and Intensive Care V, Odense University Hospital, Odense, Denmark

**Keywords:** Ambulance, Challenge, Emergency, EMS, Nepal, Prehospital, Qualitative study

## Abstract

**Background:**

Emergency medical care, including prehospital treatment, forms an important component of any healthcare system. Like most low-middle-income countries, Nepal has an emergency medical system that can be described as underdeveloped. Emergency physicians navigating this system may experience challenges or barriers in their treatment of patients. This study aimed to investigate physicians’ perspectives on emergency and prehospital patient management in a low-income country, Nepal, and to understand the challenges and barriers they perceive in emergency treatment including both the prehospital treatment and the immediate in-hospital treatment at the emergency department.

**Methods:**

Using a qualitative study, eight semi-structured interviews with physicians working in a Nepalese emergency department were performed. The interviews were conducted between September and November 2021 and were audio-recorded and transcribed verbatim. Data were subsequently analyzed using the systematic text condensation method.

**Results:**

Four main themes and associated sub-themes were identified: (1) patients’ sociocultural, educational, and financial factors (such as financial issues and financial inequality) and regional differences; (2) emergency department’s organization and resources concerning human and material resources, protocols, and guidelines; (3) problems with the emergency department (ED) service’s qualities and availability caused by an insufficient integration of the ED and the EMS, prehospital resources, and financial interests in the EMS; and (4) surrounding healthcare system’s impact on the ED where, especially, the levels of organized primary care, governmental responsibilities, and healthcare structure were addressed.

**Conclusions:**

The physicians identified numerous regularly encountered challenges and barriers. These challenges stretched beyond the ED and into various aspects of society. The patients’ financial problems were described as the greatest problem, restricting the treatment due to a given patient’s inability or unwillingness to pay for the required procedures. The physicians were thus restricted in completing their duties to the desired levels. The low quality of prehospital care and a lack of education and awareness of common diseases and symptoms in a significant proportion of patients were identified by many participants as being significant issues. The aforementioned challenges or barriers directly resulted in patients arriving in critical conditions that could have been avoided if the disease were treated earlier.

**Supplementary Information:**

The online version contains supplementary material available at 10.1186/s12245-022-00466-w.

## Introduction

Prehospital emergency medical services (EMS) form an important component of any healthcare system. The main purpose of the EMS is to stabilize patients with life-threatening conditions [1]. An effective emergency medical service system should be able to provide emergency healthcare to individuals in need of urgent medical assistance [2]. Most developed countries have an integrated EMS system that can be accessed by a single telephone number throughout the country [3, 4]. However, this is not the case in many developing countries [5]. Traditionally, emergency medical care has received only little attention when discussing improvements in healthcare systems in developing countries [1]. Considering the higher injury rates and the lesser focus on injury prevention in developing countries [6], paradoxically, this may be an area that needs thorough attention in research and healthcare interventions.

In Nepal, where the official EMS system can be described as underdeveloped and in need of improvements to provide effective emergency medical care. The current EMS system in Nepal, Nepal Ambulance Service (NAS), is a non-profit organization that was established in 2011 [7] and operates in small areas of the country. This system can be reached by a common national telephone number, 102 [7]. As described in several papers, Nepalese EMS and emergency systems face various challenges in the provision of quality emergency healthcare [8]. Numerous private ambulances are operated by various branches of government, hospitals, and non-profitable organizations [4]. All the independent operators have different ambulance phone numbers, usually the ambulance drivers’ mobile numbers, and there is no formal communication system between emergency resources (e.g., police or fire) and the EMS dispatch [8]. This leads to a decentralized EMS system with a lack of communication between organizations. This may also lead to patients being transported to facilities that may not be able to provide the needed treatment [4]. In the in-hospital setting, emergency medicine is a new specialty in Nepal having only received little attention in healthcare improvements [7]. Most challenges have been described in the setting of Kathmandu, the capital of Nepal, while few papers describe challenges in other parts of Nepal [8]. Likewise, no papers describe the in-hospital physicians’ experiences maneuvering in an emergency medical system like the Nepalese. Thus, this study aimed to investigate the physicians’ perspectives on emergency and prehospital patient management in Nepal. To our knowledge, this is the first study exploring Nepalese emergency medicine physicians’ perceived challenges or barriers in the emergency setting.

## Methods

### Study setting

Nepal is a landlocked, developing country bordering India and China. Nepal has recently been upgraded from a low-income country to a lower-middle-income country (LMIC) [9]. This study was conducted in the district of Chitwan. The capital city of the region, Bharatpur, is located in the south-central region of Nepal. Bharatpur has a population of approximately 200,000 inhabitants [10]. Our study was conducted at the College of Medical Sciences Teaching Hospital. This hospital is privately operated, and the patient treatment is fully user-payed unless the patient is covered by insurance.

In Chitwan, a single ambulance is stationed as a part of NAS [11], while the remaining ambulance system is operated by independent providers. The emergency department at the College of Medical Sciences Teaching Hospital has 23 beds, nine with patient care monitors and supplemental oxygen therapy available at the bedside, and 14 without such capabilities.

### Study design

A qualitative study was performed to elucidate the emergency physicians’ experiences and perceived challenges working in an emergency department serviced by an under-developed EMS in a developing country. The study method was based on one-on-one semi-structured interviews and conducted using a phenomenological-hermeneutic approach to gain insight into the physicians’ experiences and to understand their challenges. The authors conducted this study as part of a 6-week clinical stay at the College of Medical Sciences Teaching Hospital. To gain local perspectives and understandings of the study setting and context, the study was conducted together with local physicians. The combination of the two methodological approaches, phenomenology and hermeneutics, allowed the authors to articulate their positions during the research period and reflect on their pre-understandings.

### Data collection

We used purposeful sampling in the inclusion of emergency physicians. Purposeful sampling consists of choosing participants who are “information rich” and willing to share their experiences expressively and articulately [12]. Specifically, we chose participants with a wide range of experience to provide a variation and identify shared patterns across the participants’ statements [13]. The physicians were recruited through face-to-face conversations in the emergency department. All participants were employed at the College of Medical Sciences Teaching Hospital’s emergency department, and all were licensed to practice medicine in Nepal. The participants were all involved in clinical work at the emergency department and had a minimum of 2 months of experience at the emergency department of the College of Medical Sciences Teaching Hospital. The interviews were conducted from September 1 to November 1, 2021. They were performed face-to-face in a private office in the emergency department. The interviews were conducted in English. We developed the interview guide both inductively and deductively and combined very broad and exploring questions with more specific questions inspired by themes mentioned in other studies [4]. See Additional file 1 for the interview guide. The participants were encouraged to describe the challenges and barriers as well as their experiences, thoughts, and actions in the treatment of acute patients. The interviews were audio-recorded and transcribed verbatim by the authors with assistance from a secretary.

### Data management and analysis

The data were analyzed based on Malterud’s principles for systematic text condensation [14]. This method is developed in close relation to clinical practice and is useful for studies concerning practice-related challenges [15]. It consists of the following steps: read through the entire interview as it is while grasping the interview as a whole, go back to the beginning and read through the interview once more, trying to find “meaning units” related to the theme, put the meaning units into main groups for easier classification, and lastly, synthesize the meaning units into true statements regarding the subjective experience. Malterud’s principles are inspired by Giorgi’s method for the analysis of psychological phenomenological studies [14]. Data were analyzed individually by the authors KLH and ÅB and compared. In case of disagreement, an agreement was obtained through discussion with the senior supervisor LM. The interviews were analyzed manually by reading and rereading the interviews in Microsoft Word files (Microsoft Corporation - Redmond, Washington, USA). We identified and marked the meaning units using colored markers and used both whiteboards and paper to synthesize the meaning units during discussions between all authors. The preliminary findings were discussed in the research team during the analysis process to ensure reflection on preunderstandings and to gain a broader perspective on the material. Using the deductive-inductive method, the research team moved back and forth between the existing literature on emergency medicine in low-middle-income countries, while remaining open to new themes identified.

## Results

**Fig. 1:**
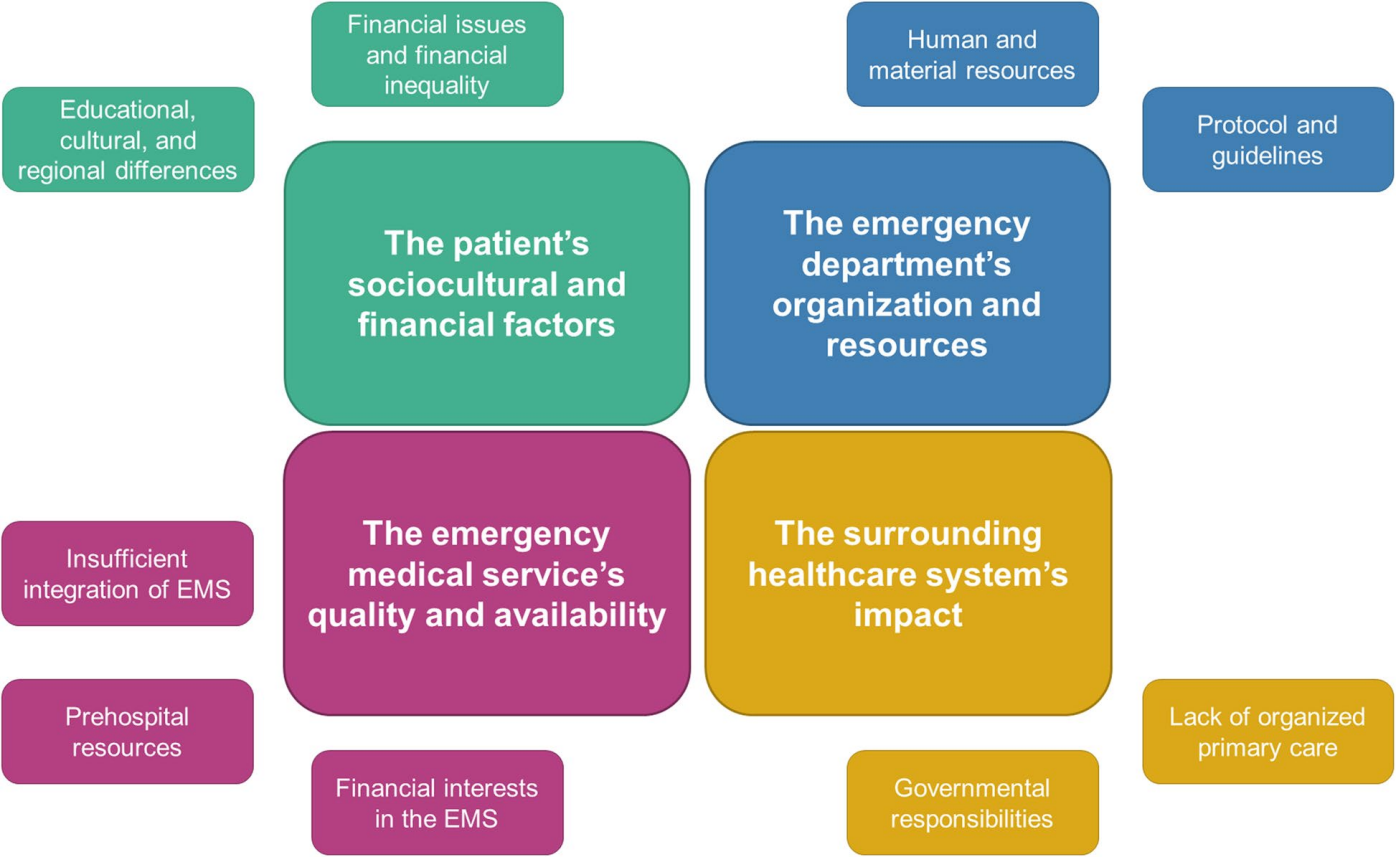
Main themes and subthemes

Eight physicians were interviewed, one female and seven males. Because of our narrow and specific aim and the in-depth nature of our interviews, the sample size was considered adequate [16]. The physicians’ experience ranged between 2 months and 11 years. Four participants were non-specialized medical officers, two were junior registrars, and two were medical officers with specializations in general practice and emergency medicine. The physicians described numerous challenges in their daily work that we divided into four main themes and associated subthemes, which are presented in Fig. 1. All themes were interrelated to various degrees and evolved around the premise that Nepal is a developing country.

### The patient’s socio-cultural and financial factors

All participants highlighted aspects of the patient’s financial, educational, and socio-cultural situations as challenging in the emergency treatment.

#### Financial issues and inequality

All participants reported that the financial difficulties of the patients were the greatest challenge or barrier to high-quality treatment. The participants described daily encounters with patients who were unable or unwilling to pay for treatment. This resulted in the discontinuation of further investigations and treatment. If patients accepted to pay for the treatment, many would get into debt as a result. The participants were frustrated by the financial aspects forming obstacles to the provision of treatment. One physician noted:

Basically, we can treat disease, we can treat disease but we can’t treat poverty. I can’t give from myself, from my pocket, I can’t spend for the benefit of the patient. That is the main challenge. (P2)

Most participants reported that some patients avoided the hospital due to a fear of the cost of the treatment. A consequence of this was that some patients only went to the hospital when the disease had progressed to a more serious stage. For example, some patients with simple conditions such as gastroenteritis were first brought to the hospital when the patient had deteriorated to reduced levels of consciousness caused by severe dehydration. Ultimately, many of such cases resulted in the death of the patient. Many participants reported that health insurance has been introduced in Nepal and considered it a great asset in combating the economic barriers to proper treatment. However, the participants reported that several patients were not aware of the government healthcare schemes or did not know how to sign up for the healthcare programs. However, the participants also described that they found it difficult to treat the richer patients as these patients expected and demanded special treatment.

#### Educational, cultural, and regional differences

The participants reported that patients not only came to the hospital with advanced stages of the disease due to a fear of the cost but also due to a lack of education regarding common diseases and symptoms. Most patients did not know when to react to symptoms and would often remain at home or seek alternative methods of treatment. Often the first line of treatment was pain management, and the physicians noted that some patients were unwilling to remain admitted to the hospital once the pain had been managed even though the underlying condition was not treated.

If you see hydronephrosis or anything like that, we will counsel them for admission, but they will say: “No, my pain has already been relieved. I want to go from the hospital and home. It has been like this for many years. Why do I need to admit [to the hospital]? Why do I need to take medicine? Why do I need to spend a lot of money?” That’s the challenge I think. (P5)

The majority of the participants described that treating patients from rural areas was challenging. The participants attributed the lack of knowledge of health issues to the level of education but also to differences in culture. Not only were rural patients more likely to be poor, but they often had a different culture regarding the management of diseases. Patients from rural areas would often seek traditional healers first. The participants were at times frustrated by these different ways of perceiving healthcare and found it challenging to inform and counsel the patients:

And as I said, in the periphery, people go to the traditional healers. And they just take the 3 grains of rice and the patient seems to be okay. Most patients in the periphery go there. I don’t know how it works, I don’t believe in that. But if we go there and tell them that this doesn’t work, they will blame us and say that we are against their traditional beliefs. (P8)

### The emergency department’s organization and resources

All participants stated that they enjoyed working in emergency medicine. They felt that their jobs were fulfilling, and they were proud of their profession. However, the structure and resources in the emergency department were described as challenging.

#### Human and material resources

Some participants described a lack of educated personnel in the ED. This led to the discontinuation of a triage system and was thus a factor in delayed diagnostics and treatment. One participant specifically mentioned that ultrasonic investigations were challenging as these were performed in a separate department and that there was a shortage of personnel to transport patients from the ED to the department of ultrasonography. Likewise, two participants pointed out that the lack of equipment to perform point-of-care investigations in the emergency department was a challenge in their daily work.

We can’t do even FAST [Focused Assessment with Sonography in Trauma] scan in the emergency ward. And we don’t have point-of-care investigations, so we need to send all the investigations to the central lab. We don’t have an emergency lab. (P4)

The lack of human resources was aggravated by a large patient flow. Several physicians described overcrowding in the ED due to a large patient flow. This led to delays in admissions and treatment. This had worsened during the COVID-19 pandemic when patients were required to have polymerase chain reaction (PCR) tests for SARS-CoV-2 performed before they could be admitted.

Sometimes there is … It’s overcrowded, which causes a lot of problems in admissions, and there is an access block upstairs because there is, like, this PCR problem. (P4)

The patients arrive from the east of Nepal and west of Nepal to here cause this is the centre. That is a big challenge because the patient flow is extremely high. (P3)

The physicians working in the ED were mostly junior doctors with limited experience in emergency medicine. The specialists in emergency medicine described having 6 months of training in emergency medicine, but lacked training in specific procedures such as, for example, handling transportation of critically ill patients from the ED to the intensive care unit:

Moving the sick patients from emergency to the ICU. Nobody is actually trained in moving. We need to have training. To move the patient, it’s kind of difficult with no training. (P4)

#### Protocols and guidelines

Many participants described the unstructured arrival of patients to the ED as challenging. Obtaining the patient's medical history had to start from scratch every time, even in cases of readmission, as there is no registry showing a patient's history. The participants reported that a triage system had existed previously in the emergency ward. This system separated the patients into zones based on how urgent they needed care. The system had been discarded due to a lack of space and resources. However, several participants described that a simpler form of triage with subjective measures to shift the patient to one of either two sides of the ER, yellow or red, had been implemented. This simpler form of triage was an adaptation of the previous system that had four zones.

Yes, big problem - then red zone. Otherwise yellow zone. It is a subjective thing. We don’t have a proper framework. But we know which case we have to shift to red zone and which case we have to shift to yellow zone. (P1)

Some participants were content with the current system, while others believed that they need a more structured system to detect critically ill patients in time. However, the participants had opposing opinions and knowledge on the current way of assessing patients. While some participants triaged patients according to a color scheme of red, yellow, and green, others reported not using any colors but instead assessed arbitrarily:

We don’t have such colour coding and triaging, but we try triaging if patient is seriously ill or if the patient is less ill. If the patient is seriously ill, requiring immediate assessment, requires monitoring, then we send the patient to one side. (P7)

If the triage doesn’t work properly, or if there is not enough manpower to do the triaging, then the needing person does not get cured properly. (P5)

However, some participants believed that the implementation of a triage system would be difficult due to a lack of knowledge.

But we have been working for it [triage system] for a long time now. But still, we are not able to establish a triage system. And we have no idea whatsoever how we will do it because we are not trained in that, no? (P4)

### The emergency medical service’s quality and availability

Most participants were positive about the number of available ambulances, and the fact that ambulances did indeed exist and could transport patients from rural and remote areas to the hospital. However, they also described challenging aspects of emergency treatment concerning prehospital systems, the available prehospital resources, and the integration and implementation of the EMS system.

#### Prehospital resources

All participants highlighted that ambulances were not well-equipped and that the prehospital personnel lacked education on how to assess and treat prehospital patients. The participants described three main types of ambulances: one purely for transport, the second type with oxygen equipment only, and the third type with more advanced equipment and health personnel. The physicians dealt with patients arriving by the first and second types of ambulances daily, but most physicians reported rarely seeing the third type of ambulance, and some even reported never having seen one. This was perceived as a major problem because critically ill patients often had not received any kind of treatment before arrival at the emergency room and could come from rural areas with long transportation times. Some described a scarcity of ambulances in rural areas compared to urban areas.

Once I admitted a patient that had travelled around 100km, no not 100km but 50km from the west. He had travelled in ambulance. The roads are very very bad. And he had travelled without oxygen. And the patient, I received him in the ER [emergency room], when I put on the oximeter, the reading showed about 40% in saturation. And I say, “Why you didn’t get ambulance with oxygen?”. He said, “No there was no ambulance with oxygen available at my place”. (P2)

Some participants described the ambulances as “taxis with sirens” and did not consider them as a part of the healthcare system. The participants were interested in increasing the quality of the ambulances and especially in having educated healthcare personnel available in the ambulances. They felt that this would have positive effects on the care.

Not like abroad, like I have seen in movies and series, there are proper paramedics that can manage the cases in between as well. But here in Nepal, there are no paramedics, no well-trained paramedics. (P1)

However, one participant noted that the implementation of more well-equipped ambulances probably would not have a large impact on the patient’s mode of transportation because the cost of these ambulances would be higher, and as such, the patients would disregard them.

#### Insufficient integration of emergency medical services

The integration of the current EMS with the hospital was described as poor by the participants. All participants reported a lack of communication between the ED and the ambulances. No mobile communication or radio communication existed between the ambulances and the ED. When patients arrived by ambulance, they were dropped off at the gate, and in-hospital personnel would take the patients from the gate into the ED. The physicians did not know if a severely ill patient was on his way and could not prepare for such scenarios. Only when the patient arrived at the gate they would know. This lack of communication was both due to the EMS providers’ lack of education and because the system was not designed to encompass this feature. The only communication physicians received from prehospital providers were from the police, who would contact the hospital in case of mass casualties.

And the negative thing is communication. We seriously lack communication. We don’t know if the patient is coming or not. Only after the patient arrives at the main gate. Only then do we get the information that the patient is coming. And we only get the information that the patient is coming. Nothing more than that. (P6)

The participants described that the patients arrived at the hospital by two means: private transportation or ambulances. Patients arriving by private means were often in less critical conditions compared to patients arriving by ambulance. The mode of transportation was influenced by the patient’s financial condition, but the participants also mentioned other reasons why patients did not utilize ambulances. The most often mentioned reason was the lack of a nationwide central emergency telephone number.

Usually, people get really panicked when they are going to call ambulances. Like a decade ago, we used to have a common number like 101 or 999. This kind of number used to be there. But right now, there are these individual clubs and you gotta become a member of a club, and get the healthcare benefits from these clubs. There is still a specific number for the government but for the private ambulances, there is no specific number. (P3)

The participants had opposing opinions and knowledge about the prehospital system. Some believed that there was a nationwide central emergency telephone number, while others did not believe that to be the case.

#### Financial interests in the emergency medical services

All participants noted that the EMS consisted of several providers: government, NGOs, and private providers. Most ambulances were believed to be run by private providers. Some participants had experienced patients who were brought to a private hospital without their knowledge or despite a specific wish to be transported to a government hospital. This influenced the treatment course, as these patients could not afford treatment at a private hospital. The physicians speculated if the ambulance drivers and the EMS providers had financial ties to specific hospitals and would get higher wages when transporting to an affiliated hospital.

But in our experience, there is a kind of syndicate. A syndicate of ambulance drivers. Even if the patient wants to go to a government hospital, they bring them to our hospital. They are unaware of it, the patients think this is a government hospital, but the ambulance drivers bring them to our hospital which is a private hospital. (P1)

Some participants were sceptical about improving the quality and the structure of the current EMS system due to the various financial interests in the service.

But it’s going to be a challenge, because like a lot of people are earning a lot of money. It’s going to be a big challenge. (P4)

### The surrounding healthcare system’s impact

The participants indicated that many of the challenges or obstacles they faced were a direct consequence of the surrounding healthcare system. They called for the government to improve the healthcare system and in turn improve the working conditions in the emergency departments.

#### Lack of organized primary care

Some participants described the lack of a well-ordered primary care system as a part of the reason why patients were more critically ill when arriving at the emergency department. They noted that patients often went to health clinics with few, or no educated healthcare professionals available, or to traditional healers. The general practitioners were not described as forming a part of the primary healthcare system, but instead, patients had the option to visit a specialist physician of their choice to attend to their symptoms. Appointments or referrals were not used. This led to patients having to wait for hours to see a physician. In some cases, the patients visited pharmacies that would prescribe antibiotics. This subsequently led to drug-resistant microorganisms. This complicated treatment in the ED as they thus often had to initiate broad-spectrum antibiotics as the initial treatment.

That is the main cause of antibiotic resistance in our country. They go to the nearest pharmacy, say, they have fever and pain abdomen and then the pharmacist prescribes antibiotics. And after that, if the symptoms don’t subside, then they come to the hospital (P8)

#### Government responsibilities

Some participants believed that the government had an unfulfilled responsibility in both the implementation of the EMS system and in addressing challenges in the healthcare system as a whole.

We are not rising the quality, we are just rising the quantity. The quality can be improved. I cannot improve the quality, I am not one in power. It should be from the governmental level, it should be from the central level. (P2)

While many acknowledged that the Nepalese government was working on improving the healthcare system, they still underscored the need for these measures to be widespread and not only focused on Kathmandu. Most participants mentioned the lack of quality in the government hospital and displayed frustration with the lack of possibilities for the poorer patient population, as these could not pay for the treatment in a private hospital, and instead received poor quality care or even no care at all:

And there is only one government hospital, Bharatpur hospital, and doctors are not available because they go to private clinics. Even if we send patients to government hospitals they return and say they can’t find doctors (P1)

## Discussion

This study found that physicians working in the emergency department in the College of Medical Sciences Teaching Hospital in Chitwan, Nepal, were generally satisfied with their job. However, the participants described several challenges and barriers in their daily work.

The physicians considered that the patients’ financial issues were a major challenge in their daily work. Nepal has a high degree of inequality with more than 8.1 million people living in poverty, and the richest 10% of the population in Nepal earning more than three times that of the poorest 40% [17]. Economic inequality presents itself in a significant geographical divide [17] where individuals with a higher degree of education live in larger cities and have a higher socioeconomic standard [18]. Most patients in low-to-middle income countries pay for healthcare out-of-pocket and, as a result, may be pushed into poverty when spending money on healthcare [19, 20]. Thus, costly healthcare deters people from using the healthcare system. This ultimately leads to worsened outcomes [21]. This translates into the emergency departments where physicians were challenged by not being able to treat patients who required treatment and being frustrated by receiving critically ill patients, who should have sought treatment earlier. The physicians mentioned that the established government-funded health insurance scheme is a possible solution to this but also highlighted that many patients are not aware of this beneficial scheme. Even though the awareness of the insurance scheme seems to be good in some areas of Nepal [22], only 11% of the population is currently enrolled in the program [23]. The patient’s reasons for not enrolling or dropping out of the health insurance scheme are various and, among others, include unfriendly healthcare workers [23]. Interestingly, the participants in this study rarely reflected upon their practice when communicating with a patient, but focused mainly on external factors as a challenge. It may create a distance between patients and physicians when physicians experience frustration with the lack of health insurance enrolment and patients are deterred from enrolment into the program due to frustrations with the physicians’ behavior. Additionally, physicians displayed frustration with the patients’ use of traditional medicine and noted that patients who had received treatment from traditional healers had delayed contact with the healthcare services and as a result received treatment too late. When trying to apply proper medical healthcare to the patients or educate the patients on the benefits of seeking professional healthcare, the physicians experienced resistance and hostility from the patients. There has not been much focus on the communication between patients and healthcare professionals in Nepal but both physicians, patients, and the healthcare system, in general, could benefit from research exploring ways of improving this.

The participants perceived that the lack of educated EMS professionals, poorly equipped ambulances, and the lack of a national universal emergency number all added to the complications experienced when admitting critically ill patients to the emergency department. In Nepal, most ambulances lack medical equipment and do not have properly educated paramedics to treat the patients, and even if patients have health insurance, this does not cover expenses related to the EMS [7]. Several studies suggest that the quality of the EMS matters when seeking to improve the healthcare systems in low-middle-income countries [4, 24–27]. The development of an EMS system is in progress in Nepal [25]. However, the quality of the ambulance services still varies greatly [28]. The physicians in this study pointed out that the EMS system was to some extent developed in Kathmandu, but also noted that this degree of development had not yet sufficiently reached other areas of Nepal. Studies from other low-middle-income countries describe the benefits of implementing “train-the-trainers” programs, World Health Organization checklists, and long-term research development [29], as well as lay first-responder units, consistency in national policies and dispatcher training programs [8, 30]. None of the aforementioned programs has been described systematically in a Nepalese context but may have the potential to form viable strategies to improve the EMS system in Nepal.

The participants in this study mentioned that the lack of communication with primary care facilities is a challenge and a barrier to the provision of healthcare of good quality. In a study concerning the healthcare systems’ preparedness for COVID-19 in Nepal, the authors concluded that the preparedness was affected by poor coordination between the different tiers, the lack of human resources, and inadequate logistic chain management [31]. These findings are in line with the findings in this study.

Emergency medicine is one of the newest recognized specialties in Nepal, and several challenges have been described in the development of the specialty such as different training procedures depending on the hospital, physician recruitment, and difficult working conditions [7]. These findings are confirmed by the respondents in our study, where a lack of human resources was described, highlighting, among others, a lack of structured training in various medical procedures. Emergency medicine is still new in Nepal, first recognized as a medical specialty in 2013. In 2015, only four individuals had completed their postgraduate training in emergency medicine in Nepal [7]. However, new programs and initiatives have been implemented in Kathmandu, where collaborative international emergency medicine training has been established [32], and new research studies have emerged describing possible points of improvement in the emergency medicine curriculum [33]. Even though research on Nepalese emergency medicine and treatment has emerged, most studies are conducted in Kathmandu with only a few studies coming from the remaining part of Nepal. Focus and implementation of research to establish and test initiatives to improve the general emergency treatment in Nepal could be beneficial to support a nationwide change and policymakers in the process.

### Strengths and limitations

Our study only included emergency department physicians working in a non-governmental hospital. This may give a one-sided view of the challenges when compared to government-funded hospitals, where the views may be different. Furthermore, this study is a single-center study, and the findings cannot necessarily be generalizable to Nepal in general. Our study does not address other perspectives on the barriers in emergency medical services, such as administrative challenges.

A strength of this study was the unstructured participant observations conducted by the interviewers 6 weeks before the interviews. This yielded knowledge of the work environment and some of the cultural aspects and strengthened the understanding of the participants.

## Conclusion

The physicians working at the emergency department identified numerous challenges, obstacles, or barriers in their work. These stretched out beyond the ED and included various aspects of society. All challenges or obstacles were interconnected and all had the potential to influence each other interchangeably. Most, if not all, were consequences of the scarce resources in the current healthcare system, poverty in the general population, and the lack of protocols, guidelines, and human resources. Together with previously published research on emergency treatment in Nepal, the challenges identified in this study offer a useful basis to inform policy and practice and improve emergency medical services.

## Supplementary Information

**Additional file 1.** Semi-structured interview guide.

## Data Availability

The data supporting this study is not available due to a risk of possibly breaching participant confidentiality.
