# Supplementary material for: Physicians’ experiences and perceived challenges working in an emergency setting in Bharatpur, Nepal: a qualitative study
Source: Int J Emerg Med. 2022 Nov 8;15:61. doi: 10.1186/s12245-022-00466-w (PMC9643944; doi:10.1186/s12245-022-00466-w)
Supplement: Supplementary file 1 — Additional file 1. Semi-structured interview guide. [file 12245_2022_466_MOESM1_ESM.pdf]

Additional File 1: Semi-structured interview guide

**Introduction**	
	What is your profession?<br>Are you a specialist in your field, or still under specialization?<br>Where did you get your education?<br>How long have you worked as a doctor?<br>How long have you worked in the emergency medical ward here?<br>Have you ever worked abroad? If so, where, and how long?
**Main questions**	
**Experiences/Challenges**	How do you experience the arrival of patients in the emergency ward?<br>Do you experience any challenges in the admission process of patients? *If so, how?*<br>What are your experiences with ambulance services in your daily work?<br>How would you describe your teamwork with prehospital providers?<br>Do you experience any challenges with the way patients are admitted to the emergency room? *If yes, which?*
**System**	What are the steps of a typical admission process in your emergency department?<br>Do you have any sort of triage system for patients? If yes, can you describe it to me?<br>How do you experience this process (triage process)?<br>How do you experience challenges in the admission process?<br>What are your experiences with critically ill patients and possible treatment before arriving at the hospital?<br>Where do patients, in general, seek health advice first? Do patients, in general, come directly to you, or seek other health care services first?
**Improvement**	Do you think it is possible to implement a full-scale ambulance service in Bharatpur, and Nepal as a whole? If yes, how so? If no, why not?<br>If any changes/improvements could be made, which ones do you think would benefit patients the most?
	**Closing**

	Do you enjoy working here?<br>What is the thing you enjoy the most about your work?<br>Is there anything you would like to add?
